# Effects of Mixing and Large-Amplitude Oscillatory Shear Deformations on Microstructural Properties of Gliadin and Glutenin as Captured by Stop-Flow Frequency Sweeps in Small-Amplitude Oscillatory Shear †

**DOI:** 10.3390/foods13203232

**Published:** 2024-10-11

**Authors:** Gamze Yazar, Brennan Smith, Jozef L. Kokini

**Affiliations:** 1 Food Science Department, Purdue University, 745 Agriculture Mall Dr., West Lafayette, IN 47907, USA; 2 Department of Animal, Veterinary and Food Sciences, University of Idaho, 875 Perimeter Dr. MS 2312, Moscow, ID 83844, USA; brennan.smith@usda.gov; 3 USDA-ARS-SRRC Food Processing and Sensory Quality, 1100 Allen Toussaint Blvd., New Orleans, LA 70124, USA

**Keywords:** gliadin, glutenin, Farinograph mixing, rheology, viscoelastic properties

## Abstract

Gliadin and glutenin extracted from vital wheat gluten were studied using Large Amplitude Oscillatory Shear (LAOS) followed by stop-flow frequency sweep tests after being subjected to short (4 min) and prolonged (60 min) mixing times. The LAOS tests were conducted at up to two different strain amplitudes (γ: 0.1%, 200%; *ω*: 10 rad/s) to apply small and large deformations to the gliadin and glutenin after mixing for different time periods. Frequency sweep tests (*ω*: 0.01–100 rad/s, γ: 0.06%) revealed an increase in the elasticity of gliadin with respect to an increasing mixing time, as evidenced by a robust increase in G′(*ω*), coupled with a less robust increase in G″(*ω*). Consistent with the increase in elasticity, a progressively lower tanδ(*ω*) and G′(*ω*) slope were observed for the gliadin that underwent 60 min of mixing followed by large LAOS deformations. However, G′(*ω*), G″(*ω*), and η*(*ω*) remained constant for glutenin as the mixing time increased. Elastic decay with an increase in tanδ(ω) was found for glutenin when subjected to prolonged mixing followed by large LAOS deformations, which became apparent at high frequencies. The stop-flow LAOS (non-linear region)–frequency sweep (linear region) tests provided an understanding of how exposure to different mixing times and LAOS deformations of different magnitudes influence the mechanical/rheological properties of the main gluten proteins.

## 1. Introduction

The economic importance of wheat and its contribution to the diet of humans and livestock cannot be disputed [1]. Wheat flour and wheat-based products, such as pasta, bread, and other bakery products, are considered essential for human nutrition worldwide, since they are important sources of macronutrients (mainly carbohydrates and protein), micronutrients (vitamins and minerals), dietary fiber, and antioxidants [2]. Considering the increasing trend towards a plant-based diet in recent years [3], the demand for wheat production could become even higher. The currently available data indicate an increase in the average annual global production of wheat in 2024/2025, that is 791.4 million tons. This makes wheat the second-most important cereal, following coarse grains with an estimated annual global production of 1523 million tons over the same period [4].

Determining the mechanical behavior of wheat flour is crucial in the food industry for ensuring consistent end-product quality. This quality can vary significantly due to fluctuations in wheat quality related to genetic factors and environmental changes [5,6]. Dough processing operations apply different forms of deformations with a wide range of magnitudes on dough. The quality of the final product is greatly affected by the specific physicochemical changes that the wheat flour dough experiences at each processing stage [7,8]. During mixing, which is the first step of dough processing, the mechanical energy applied, along with the hydration of the flour particles, promotes the crosslinking of gliadins and glutenins, resulting in the formation of a continuous protein network [9]. Studies based on fundamental rheological methods have been conducted to explore how wheat flour macromolecules, mainly gluten proteins, interact under mechanical mixing forces and after the removal of these mixing forces. Cuq et al. [10] used a capillary flow rheometer to study the shear and extensional flow properties of wheat flour doughs as a function of the mixing time (3–60 min) and the rest time (0 or 120 min) after mixing. Kim et al. [7] conducted stress growth tests to determine the impact of the mixing time and resting on the non-linear rheological properties of strong and weak hard wheat flour doughs. The impact of different times of Farinograph mixing on the non-linear viscoelastic responses of hard wheat flour dough [8] and soft wheat flour dough [11] were studied using the LAOS technique (γ: 0.01–200%; ω: 0.1, 1, 10, 20 rad/s). Bonilla et al. [12] studied the distribution of gliadin, high molecular weight (HMW) glutenins, and low molecular weight (LMW) glutenins in different doughs (soft wheat, hard wheat, and semolina flour doughs) during the different stages of mixing through a novel ‘*in-situ*’ detection and quantitative imaging techniques coupled with small-amplitude oscillatory shear tests. Vidal et al. [9] developed another in-line method based on consecutive stress relaxation steps with an increasing deformation to study the mixing process of wheat flour dough from a rheological standpoint, while eliminating the sample transfer from the mixer to rheometer. These studies have shown that the mechanical properties of wheat flour dough greatly depend on the mixing time, the following resting time, and the redistribution of the gluten proteins during these processing steps.

In most dough processing operations, mixing is followed by resting or bulk fermentation, sheeting, sizing, shaping, or molding before proofing and/or baking [6,13]. As the processability of wheat flour into dough and baked goods largely depends on its gluten proteins [12,14,15], and considering the deformations applied to dough during and after mixing, the characterization of the linear and non-linear viscoelastic properties of gluten networks has always been of interest. The aim of this fundamental study was to discover more detailed insights into the changes experienced by gluten proteins under deformations of various magnitudes. Therefore, the impact of mixing and the following LAOS deformations on the microstructural properties of the main gluten proteins, gliadin and glutenin, were studied. The changes in the microstructures of the gluten proteins resulting from the mechanical mixing forces of a Farinograph mixer for different time periods, and the increasing strain amplitudes during the LAOS tests were probed by sudden stop-flow LAOS–frequency sweep experiments, which captured a snapshot of the microstructures after large deformations. The LAOS tests described the material behavior during and after the transition from a linear to non-linear viscoelastic region by gradually increasing the amplitude of the strain, while the non-destructive small-amplitude oscillatory shear (SAOS) tests, such as the frequency sweep tests, analyzed the linear viscoelastic response to small strains without disturbing the 3D structure of the materials [16]. The stop-flow LAOS-SAOS tests used in this study enabled us to understand the impact of mixing and the following deformations on the gliadin and glutenin microstructures, and the individual responses of these gluten proteins to the changing deformations in a more detailed and informative manner. Thus, the synergistic effect of these proteins on dough development and their sensitivities to varying deformations, such as those applied during dough processing, are better understood.

## 2. Materials and Methods

### 2.1. Materials

Vital wheat gluten (Gluvital^TM^ 21020) was purchased from Cargill (Düsseldorf, Germany) and 95% ethanol (*v*/*v*) was purchased from PHARMCO-AAPER (Brookfield, CT, USA). The vital wheat gluten had 75.8% protein, 0.9% ash, 7.4% lipids, 6.4% moisture, and residual starch of around 10%, as previously reported by [17].

### 2.2. Methods

#### 2.2.1. Extraction of Gluten Proteins

Gliadin and glutenin were extracted from the vital wheat gluten (VWG) using Osborne fractionation [18]. The VWG was washed with 70% ethanol (*v*/*v*) with a two-step extraction. The ratio of the VWG to 70% ethanol was 1:5 (*w*/*v*) in each step. After washing the VWG with ethanol for 24 h, the mixture was centrifuged at 10,000 rpm for 10 min at room temperature. The supernatant that contained the gliadins and the precipitant, including the glutenins, was collected, freeze-dried, and ground into powder for further analysis.

#### 2.2.2. Farinograph Mixing

The moisture contents of the extracted gliadin and glutenin were determined according to the AACC method 44-15.02 [19]. Depending on the moisture level, appropriate amounts of the samples were weighed (on a 14% moisture basis). Farinograph tests (AACC method no. 54-21.02) were performed using a Brabender Farinograph (Duisburg, Germany). The other aspects of similar experiments have been discussed before [20]. Both gluten proteins were mixed at their optimum water absorption capacities for 4 min and 60 min.

#### 2.2.3. Rheological Tests

The LAOS measurements were conducted using a DHR-3 Rheometer (TA Instruments, New Castle, DE, USA). The non-linear properties of the gliadin and glutenin were tested at strains ranging from 0.01% to 200%. Prior to the LAOS testing, the samples were rested until their axial normal forces reached a value below 1 N. All the LAOS data were collected at a frequency of 10 rad/s at 25 °C. A 20 mm x-hatch parallel-plate geometry and a gap of 2 mm were used [15]. The measurements were conducted in triplicate and the averages of the oscillatory stress response data were calculated using Fourier transforms. The LAOS parameters used to characterize the non-linear viscoelastic properties of the gluten proteins in this study have been previously defined [16,21,22].

The impact of the LAOS deformation on the structural properties of the gliadin and glutenin was evaluated through frequency sweep tests. Oscillatory shear deformation tests were conducted at up to 0.1% (SAOS) and 200% (LAOS), and then stop-flow frequency sweep tests in the SAOS region were applied immediately after the strain sweep was stopped. A strain value of 0.06% was selected for conducting the frequency sweep tests in the linear region. A frequency range of 0.01 rad/s to 100 rad/s was used and the tests were conducted at 25 °C. The geometry settings remained the same as for the LAOS tests. The SAOS tests were conducted in triplicate and the average values were reported. The SAOS parameters obtained through frequency sweeps to determine the linear viscoelastic properties were defined in an earlier study [21].

All the linear and non-linear data were analyzed using TRIOS software (version 4.4.0.41651) and plotted using OriginPro 8.6.

#### 2.2.4. Statistical Analysis

A 95% confidence level was used for the statistical analyses conducted in OriginPro 8.6. To compare the data obtained for the gliadin and glutenin, a one-way ANOVA with Tukey’s comparison test (*p* < 0.05) was used. The rheology data were compared separately at each LAOS strain. Letters were used to denote the significant differences in the mean values across the samples.

## 3. Results and Discussion

### 3.1. Farinograph Mixing Behavior

**Figure 1:**
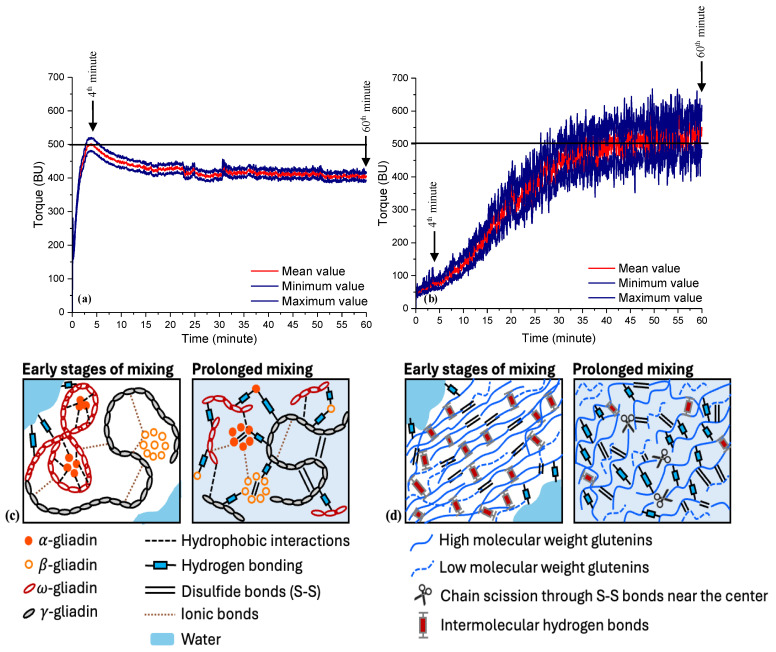
Farinograms for gliadin (**a**) and glutenin (**b**). Mean value indicates average consistency based on minimum and maximum values. Schematic illustrations of how gliadins (**c**) and glutenins (**d**) interact during mixing are given below Farinograms.

The Farinograms obtained for the gliadin and glutenin after 4 and 60 min of Farinograph mixing are shown in Figure 1. These Farinograms unveil distinct differences in the mixing properties of the gliadin and glutenin. The gliadin shows a development peak in the early stages of mixing that is typically observed in wheat flour doughs, while the glutenin displays a gradual development without the presence of a peak throughout the 60 min Farinograph mixing. The development time, at which the center of the mixing curve reaches the 500 BU consistency line, is 3.3 ± 0.5 min for the gliadin and 31.4 ± 0.3 min for the glutenin.

The Farinograph results here (Figure 1) indicate that gliadin development contributes to the overall peak development much earlier than that of glutenin. Glutenin appears to contribute to dough strength as mixing proceeds. A higher glutenin-to-gliadin ratio in wheat flours has been reported to result in an increase in mixing time [23,24], which is consistent with the extended development time found for glutenin in this study (Figure 1b).

During the Farinograph mixing, the consistency of the gliadin first increased due to the hydration-induced protein–protein interactions up to around 4 min, where a peak occurred. Then, continued mixing resulted in a decay in the torque values, after which the consistency remained almost stable during prolonged mixing (Figure 1a). The gliadin’s mean consistency was stable at around 415 BU for mixing times ranging from 22.75 min to 50.9 min, then dropped to 403.5 ± 23.3 BU at the end of the Farinograph mixing. The mixing process is divided into three distinct stages: the distribution of materials, hydration, and energy input [25]. Certain monomeric proteins, such as α-gliadins and ω-gliadins, aggregate by hydrophobic interactions until optimum mixing (peak point), and then they disintegrate with continued mixing [26,27], as shown in Figure 1c, which is consistent with the increasing consistency of the gliadin until it started to decrease beyond the peak point (Figure 1a). The use of circular dichroism spectroscopy to study the secondary structures of gliadins revealed that ω-gliadins are rich in randomly coiled β-turns stabilized by strong hydrophobic interactions, without a detectable α-helix or β-sheet, while α/β- and γ-gliadins contain 30–35% α-helix and 10–20% β-sheet conformations [28]. ω-gliadins were reported to have rod-like extended structures that are greater in size when compared to those of globular α-gliadins. The differences in their secondary structures, shapes, and sizes suggested different roles for ω- and α-gliadins during dough formation [29]. During the energy input phase of dough mixing, protein molecules are stretched and aligned as they are subjected to shear and uniaxial extension deformations through an applied mechanical energy [6,30,31]. The stretching events applied during nanomechanical force tests pointed to a more compact structure, even at 8M urea, for the β-turn-rich ω-gliadins, while the more globular α-gliadins unfolded at 2M urea [29], supporting the dissociation of gliadin sub-units under continued mixing forces [26,27], as reflected in the Farinogram obtained for gliadin in the current study (Figure 1a). Besides the hydrophobic interactions that have been suggested to occur between ω- and α-gliadins, α/β- and γ-gliadins are stabilized by covalent disulfide bonds and noncovalent hydrogen bonds in their α-helices and β-sheets [28]. The hydrogen bonds in dough are known to be much weaker than the disulfide bonds; however, they play an important role in defining the rheological behavior of gluten networks due to their ability to interact under stress, and thus facilitate the re-orientation of gluten proteins. The functionality of the hydrophobic interactions occurring between non-polar groups of gliadin in the presence of water is similar to that of hydrogen bonds, but the overall effect is much smaller [32]. Therefore, the stabilization of gliadin’s consistency under continued Farinograph mixing forces can be mainly attributed to the presence of hydrogen bonds in gliadin.

As for glutenin, the constantly developing mixing curve appeared to stabilize at 500 BU after 31 min of mixing, with 100.6% of added water into dry glutenin on a weight basis (Figure 1b). Even though the consistency increased somewhat as mixing continued, the final mean consistency was 512.5 ± 26.1 BU after 60 min of mixing. When the added water amount was lower, a 500 BU consistency was reached earlier, but the stabilization of the mean value of the mixing curve occurred at higher consistencies. The opposite was observed when higher levels of water were added. Thus, the water absorption capacity (WAC) of glutenin depends on the stabilization of the mixing curve at a 500 BU consistency.

The cysteine residues in glutenin can form inter- and intrachain disulfide bonds, leading to the formation of a highly networked structure [33]. During mixing, oxygen is incorporated in the dough and helps strengthen the dough, contributing to the formation of S-S bonds [30]. High molecular weight (HMW) and low molecular weight (LMW) glutenin subunits are crosslinked through cysteines forming intermolecular disulfide bonds (Figure 1d), and thus contribute to network formation [33]. The length of the linear HMW glutenin molecules determines the degree of polymer–polymer interactions and the extent of the network being formed [34], as HMW glutenins are regarded as the backbone of a gluten network [12]. FTIR spectroscopy results led Belton [34] to develop the train (polymer–polymer interaction)–loop (water–polymer interaction) model to explain the interactions among glutenins in dough. According to this model, in the absence of water, glutenin chains bind to each other via intermolecular hydrogen bonds to form a dense mass. The number of water–protein hydrogen bonds increases with the addition of water, as shown in Figure 1d. Upon hydration, the quantity of the β-sheet structures in glutenin first increases and then decreases, which is associated with the ordering resulting from the transition from a glassy state to rubbery region [35]. As hydration proceeds, the extent of the train region in the β-sheet conformation decreases and that of the hydrated loop region increases [34]. These results are consistent with the Farinographs showing the consistency of glutenin (Figure 1b). In addition to the decrease in the train regions, chain scission through the S-S bonds formed near the center of the large glutenin molecules [36] and the detachment of LMW glutenins from the backbone due to continued mechanical forces [12] are the reasons behind the decrease in the rate of increase in the consistency of glutenin when subjected to prolonged mixing (Figure 1d). On the other hand, the absence of polymerization terminators, such as gliadins with an odd number of cysteines and glutathione [33,37], results in a somewhat increasing consistency even during prolonged mixing (Figure 1b), which is the opposite of what we observed for gliadin (Figure 1a).

The amounts of water used to obtain these mixing profiles for gliadin and glutenin were 68% (34 mL water for 50 g of gliadin, adjusted on a 14% moisture basis) and 100.6% (50.3 mL water for 50 g of glutenin, adjusted on a 14% moisture basis), respectively. The added water levels for the mixing of these gluten proteins reveal the significantly higher water absorption capacity of glutenin. The presence of gliadin and glutenin together creates a synergy with regard to the water absorption capacity, which is not possible to explain with the WAC of gliadin or glutenin alone. The amount of water added for the Farinograph mixing of the gluten was 63.5 mL per 50 g gluten (on a 14% moisture basis) [17], suggesting an increase in the WAC of gliadin and glutenin when linked together to form a gluten network. The consistency of the gluten was stable once 500 BU was reached [17]. The slightly decreasing consistency for gliadin (Figure 1a), and the slightly increasing consistency found for glutenin (Figure 1b), with prolonged mixing, explains the balancing contribution of both gliadin and glutenin to the stable consistency of gluten, as suggested by Yazar et al. [17].

### 3.2. Analysis of Stop-Flow LAOS–Frequency Sweeps for Gliadin Obtained at Different Mixing Times

**Figure 2:**
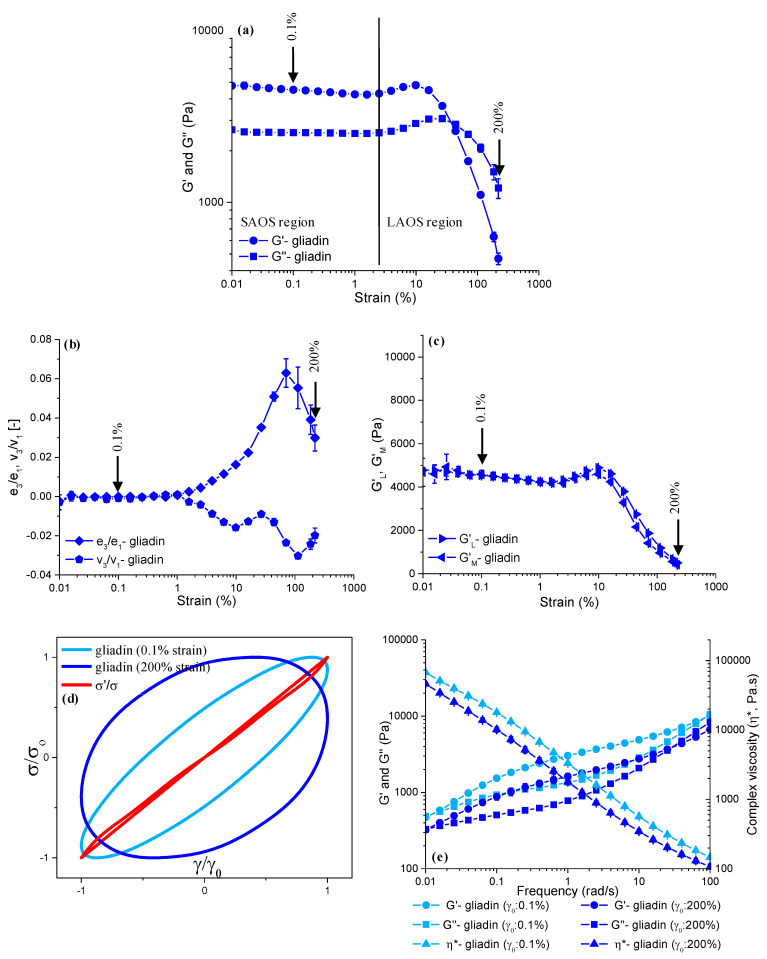
LAOS parameters and frequency sweep data for gliadin obtained at **4 min** of Farinograph mixing (γ: 0.1%, 200%): (**a**) LAOS sweeps, (**b**) the ratio of the third-order to the first order elastic (e_3_/e_1_) and viscous (v_3_/v_1_) Chebyshev coefficients, (**c**) large strain and minimum strain moduli (G_L_, G_M_), (**d**) elastic Lissajous-Bowditch curves at γ: 0.1% and 200%, (**e**) frequency sweeps.

**Figure 3:**
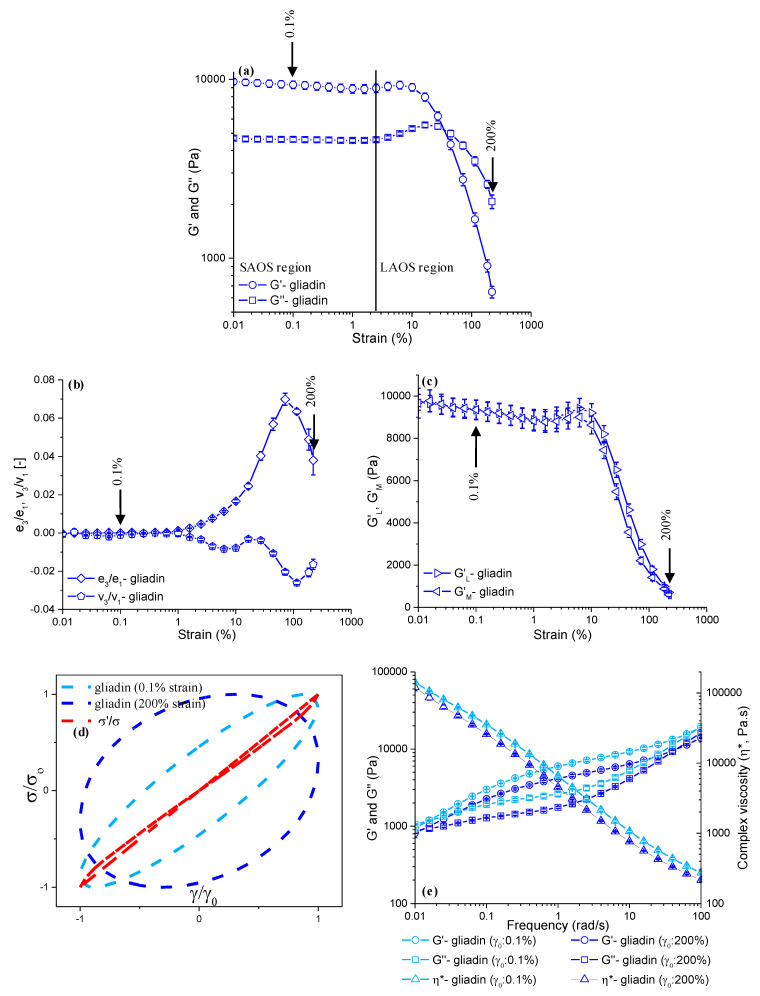
LAOS parameters and frequency sweep data for gliadin obtained at **60 min** of Farinograph mixing (γ: 0.1%, 200%): (**a**) LAOS sweeps, (**b**) the ratio of the third-order to the first order elastic (e_3_/e_1_) and viscous (v_3_/v_1_) Chebyshev coefficients, (**c**) large strain and minimum strain moduli (G_L_, G_M_), (**d**) elastic Lissajous-Bowditch curves at γ: 0.1% and 200%, (**e**) frequency sweeps.

The impacts of short-term (4 min) mixing (Figure 2) and prolonged (60 min) mixing (Figure 3), both followed by LAOS deformations of up to a γ_0_ of 0.1% and a γ_0_ of 200%, on the microstructure of gliadin were evaluated through the strain sweep data (G′ and G″), LAOS parameters (elastic and viscous Chebyshev coefficients [e_3_/e_1_, v_3_/v_1_], large and minimum strain moduli [*G′_L_*, *G′_M_*]), elastic Lissajous–Bowditch curves, and frequency sweep data (G′, G″, η*, and tanδ).

The gliadin that underwent 4 min of Farinograph mixing had G′ values of 4525 ± 129.8 Pa at a γ_0_ of 0.1%, and 469 ± 36.3 Pa at a γ_0_ of 200% (Figure 2a), indicating an order-of-magnitude decay in elasticity as the gliadin entered the LAOS region. When the gliadin was subjected to 60 min of mixing (Figure 2a), the G′ values at a γ_0_ of 0.1% and 200% were almost twice as high as those found for the gliadin mixed for 4 min (Figure 3a). Thus, the LAOS sweeps indicated the more elastic behavior of gliadin in the linear (γ_0_: 0.1%) and non-linear regions (γ_0_: 200%) as the mixing time increased. The difference between the G′ of the gliadins mixed for 4 min (Figure 2a) and 60 min (Figure 3a) started to decline as the LAOS deformation increased; however, it was still significant (*p* < 0.05) at a γ_0_ of 200%. The G′ values of gliadin for an increasing mixing time showed the opposite trend of what was observed for the Farinograph consistency of gliadin (Figure 1a). The reason behind the increase in G′ with respect to an increasing mixing time (Figure 2a and Figure 3a), while the Farinograph consistency decreased (Figure 1a), could be attributed to the induced gliadin–gliadin interactions and the restoring ability of gliadin during the resting phase between mixing and the LAOS testing [38,39].

Both gliadins obtained after 4 min and 60 min of mixing displayed G′ and G″ overshoots at the onset of non-linearity beyond a strain value of 1.5% (Figure 2a and Figure 3a), which was regarded as the critical strain (γ_cri_) for gliadin, as suggested in an earlier study [20]. The overshoots observed for the G′ and G″ values of gliadin, regardless of the mixing time, were indicative of type IV non-linear behavior according to the classification by Hyun et al. [40]. As the amplitude of the strain increased into the LAOS region, a crossover point (G′ = G″) was observed at around a γ_0_ of 40% for the gliadins obtained at both mixing times, indicating that the viscous response started to dominate the viscoelastic behavior of gliadins under LAOS deformations.

The elastic (*e*) and viscous (*v*) Chebyshev coefficients were evaluated to obtain a detailed understanding of how mixing and LAOS deformations affected the gliadin and glutenin within each oscillatory cycle. The emergence of third-order harmonics has been associated with the presence of non-linearity [41]. Therefore, the ratios of the third-order elastic (*e_3_*) and viscous (*v_3_*) Chebyshev coefficients to the first-order Chebyshev coefficients (*e_1_*, *v_1_*) were evaluated. The *e_3_*/*e_1_* and *v_3_*/*v_1_* values remained marginal up to a γ_0_ of 1.5% for the gliadins mixed for both 4 min (Figure 2b) and 60 min (Figure 3b), suggesting a linear viscoelastic response below this strain amplitude, as previously reported by Ewoldt et al. [22]. This result is in accordance with the critical strain indicated by the G′ and G″ data (Figure 2a and Figure 3a). As the applied LAOS deformation increased beyond 1.5%, the *e_3_*/*e_1_* values started to increase, showing a peak maximum at a γ_0_ of 70%, indicating the intracycle strain-stiffening behavior (*e_3_*/*e_1_* >0) of gliadin regardless of the mixing time. The magnitude of *e_3_*/*e_1_* at a γ_0_ of 70% was 0.062 ± 0.007 for the gliadin mixed for 4 min (Figure 2b), while it was similar (*p* > 0.05) for the gliadin obtained after 60 min of mixing, with a magnitude of 0.069 ± 0.003 (Figure 3b). The same similarity was also obtained for the *e_3_*/*e_1_* values of the gliadins obtained at both mixing times (*p* > 0.05) at a γ_0_ of 200%, indicating that long mixing times did not cause a significant change in the intracycle strain-stiffening of the gliadin as the amplitude of the strain increased. In earlier studies, the origin of the strain-stiffening behavior of wheat flour dough was reported to be due to the entanglements of large glutenin molecules [42]. This study reveals that gliadin also shows strain-stiffening behavior when subjected to large deformations.

As for the viscous measures, the third harmonic viscous Chebyshev coefficients normalized by the first harmonic coefficients (*v_3_*/*v_1_*) were negative in the non-linear viscoelastic region for both the gliadins obtained at 4 min (Figure 2b) and 60 min of mixing (Figure 3b), suggesting that the intracycle shear-thinning behavior, as *v_3_*/*v_1_* < 0, is associated with intracycle shear-thinning [22]. Similarly, intracycle shear-thinning behavior in the non-linear region has been reported for gliadin in other studies [15,20]. The lowest *v_3_*/*v_1_* values found here were for a γ_0_ of 110%, with magnitudes of −0.030 ± 0.00 (Figure 2b) and −0.026 ± 0.00 (Figure 3b) for the gliadins subjected to 4 min and 60 min of mixing, respectively. The significantly higher *v_3_*/*v_1_* values (*p* < 0.05) obtained for the gliadin mixed for 60 min are indicative of a lower degree of intracycle shear-thinning behavior due to the prolonged mixing.

The large strain modulus (*G′_L_*) and minimum strain modulus (*G′_M_*) for the gliadin obtained at 4 min of Farinograph mixing and then subjected to a 0.1% strain amplitude remained almost equal (*p* > 0.05) (Figure 2c), which was because this strain amplitude was within the limits of the linear viscoelastic region for gliadin, as previously observed from the strain sweep data (Figure 2a) and Chebyshev coefficients (Figure 2b). At a γ_0_ of 200%, the *G′_L_* (500 ± 43.5 Pa) for gliadin was found to be slightly higher than the *G′_M_* (412 ± 86.0 Pa), indicating intracycle strain-stiffening behavior [22] for the gliadin that underwent 4 min of Farinograph mixing. On the other hand, the *G′_L_* and *G′_M_* values at a γ_0_ of 0.1% for the gliadin obtained at 60 min of Farinograph mixing (Figure 3c) were twice the values of the gliadin subjected to 4 min of mixing (Figure 2c) followed by LAOS deformations of up to γ_0_ ≤ 0.1%, suggesting a much stiffer system in the linear region for gliadin with prolonged mixing. When a 200% LAOS strain was applied, the *G′_L_* was 700 ± 60.1 Pa and the *G′_M_* was 597 ± 86.7 Pa for the gliadin mixed for 60 min, indicating intracycle strain-stiffening behavior, as was the case for the gliadin subjected to 4 min of mixing. These data concur with the strain sweep data (Figure 2a and Figure 3a), where the decay observed for the G′ and G′′ values is in line with the sharper decay in the G′_L_ and G′_M_ for the gliadin that underwent prolonged mixing during the transition from the linear (γ_0_: 0.1%) to non-linear region (γ_0_: 200%), compared to that found for the gliadin after 4 min of mixing.

The normalized elastic Lissajous–Bowditch curves of gliadin at a γ_0_ of 0.1% and a γ_0_ of 200% are shown in Figure 2d and Figure 3d. Once the gliadins obtained at both mixing times were subjected to small deformations (γ_0_: 0.1%), the normalized elastic Lissajous–Bowditch curves displayed narrow elliptical trajectories (Figure 2d and Figure 3d), which is an indication of linear viscoelastic behavior [43]. As the applied LAOS strain increased from 0.1% to 200%, the elastic Lissajous–Bowditch curves showed wider trajectories for the gliadins that underwent both short and prolonged mixing times, suggesting more viscous-like viscoelastic behavior compared to that found in the linear region.

The frequency sweeps for the gliadins obtained at 4 min (Figure 2e) and 60 min (Figure 3e) of Farinograph mixing were then subjected to 0.1% and 200% LAOS strains. The G′(ω) recorded at the lowest frequency (0.01 rad/s) was significantly lower than that obtained at the highest frequency (100 rad/s), for both applied LAOS strains of 0.1% and 200%. In comparison to 4 min of mixing (Figure 2e), 60 min of Farinograph mixing resulted in higher G′(ω) values throughout the whole frequency range (*p* < 0.05) (Figure 3e). Except for the G′(ω) of the gliadin that underwent 60 min of mixing at 0.01 rad/s, the stop-flow frequency sweep data point to a decrease (*p* < 0.05) in G′(ω) as the applied LAOS strain increases from 0.1% to 200%, causing a transition from the linear to non-linear region, and indicating the elastic decay of the gliadin. This result concurs with the elastic Lissajous curves (Figure 2d and Figure 3d), as they also show a sharp increase in the loop area as the strain increases from 0.1% to 200%. Although the Lissajous–Bowditch curves become wider as the LAOS strain increases for the gliadins for both mixing times, the reason behind the insignificant effect of increasing the LAOS strain (*p* > 0.05) on the G′(ω) of the gliadin mixed for 60 min at 0.01 rad/s (Figure 3e) is associated with the increase in the elastic response of gliadin with prolonged mixing. Increasing the mixing time for gliadin from 4 min to 60 min causes the G′(ω) to increase, revealing an increase in the elasticity of gliadin as the mixing time increases (Figure 2e and Figure 3e).

The gliadin showed two crossover points (G′ = G″) for the applied frequency range, and the frequencies at which the crossovers occurred were affected by the mixing time. The crossover frequency is an important parameter for defining the viscoelastic character of materials, as it indicates the transition from an elastic solid (G′ > G″) to a viscous liquid (G″ > G′), or vice versa [40]. The presence of two crossover frequencies for gliadin indicated viscous-like behavior (G″ > G′) at very low frequencies, which transformed into elastic-like behavior (G′ > G″) as the frequency increased, and then again into viscous-like behavior (G″ > G′) at very high frequencies. When the gliadin was mixed for 4 min and then subjected to 0.1% and 200% of LAOS strains, the crossover points were observed at 0.012 rad/s and 85 rad/s, and at 0.01 rad/s and 29 rad/s on the frequency sweeps, respectively, indicating reduced resistance to increasing frequencies with exposure to high LAOS strains. As the mixing time increased to 60 min, the crossover points shifted to higher frequencies. The gliadin subjected to a 0.1% LAOS strain displayed crossovers at 0.014 rad/s and 88 rad/s, while that subjected to a 200% LAOS deformation had crossover points at 0.012 rad/s and 45 rad/s, suggesting the improved resilience of gliadin with prolonged mixing to increasing frequencies. The complex viscosity [η*(ω)] values increased (*p* < 0.05) as the mixing time of the gliadin increased from 4 min (Figure 2e) to 60 min (Figure 3e), concurring with the crossover points and strain sweep data (Figure 2a and Figure 3a). Increasing the LAOS deformation from 0.1% to 200% resulted in a significant decrease (*p* < 0.05) in the η* values of the gliadin samples obtained at both 4 min and 60 min of Farinograph mixing, suggesting a decay in the linear viscoelastic behavior of gliadin within the applied frequency range (0.01–100 rad/s).

### 3.3. Analysis of Stop-Flow LAOS–Frequency Sweeps for Glutenin Obtained at Different Mixing Times

**Figure 4:**
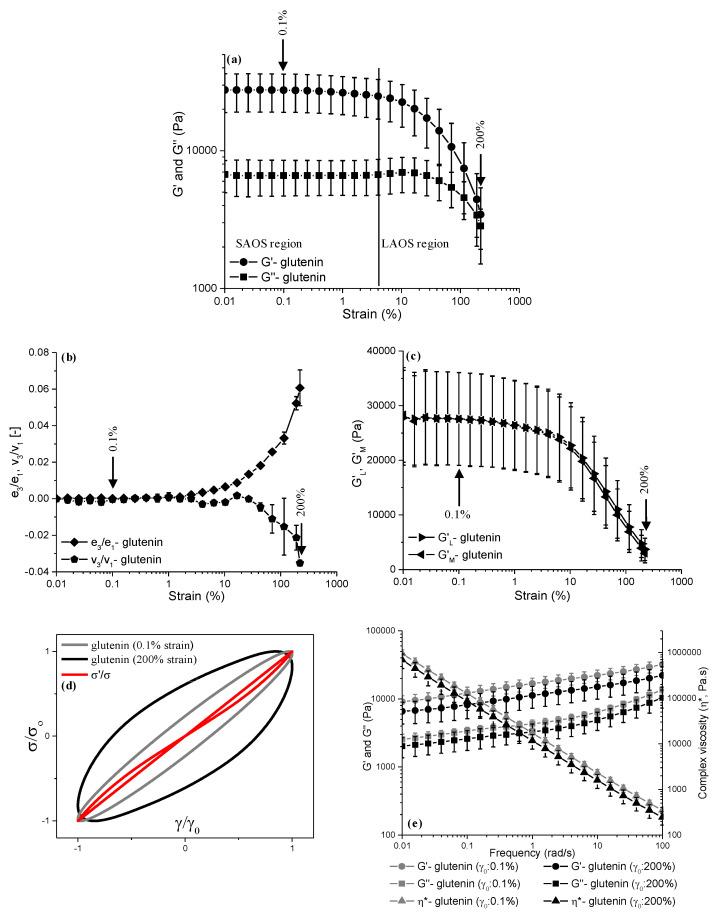
LAOS parameters and frequency sweep data for glutenin obtained at **4 min** of Farinograph mixing (γ: 0.1%, 200%): (**a**) LAOS sweeps, (**b**) the ratio of the third-order to the first order elastic (e_3_/e_1_) and viscous (v_3_/v_1_) Chebyshev coefficients, (**c**) large strain and minimum strain moduli (G_L_, G_M_), (**d**) elastic Lissajous-Bowditch curves at γ: 0.1% and 200%, (**e**) frequency sweeps.

**Figure 5:**
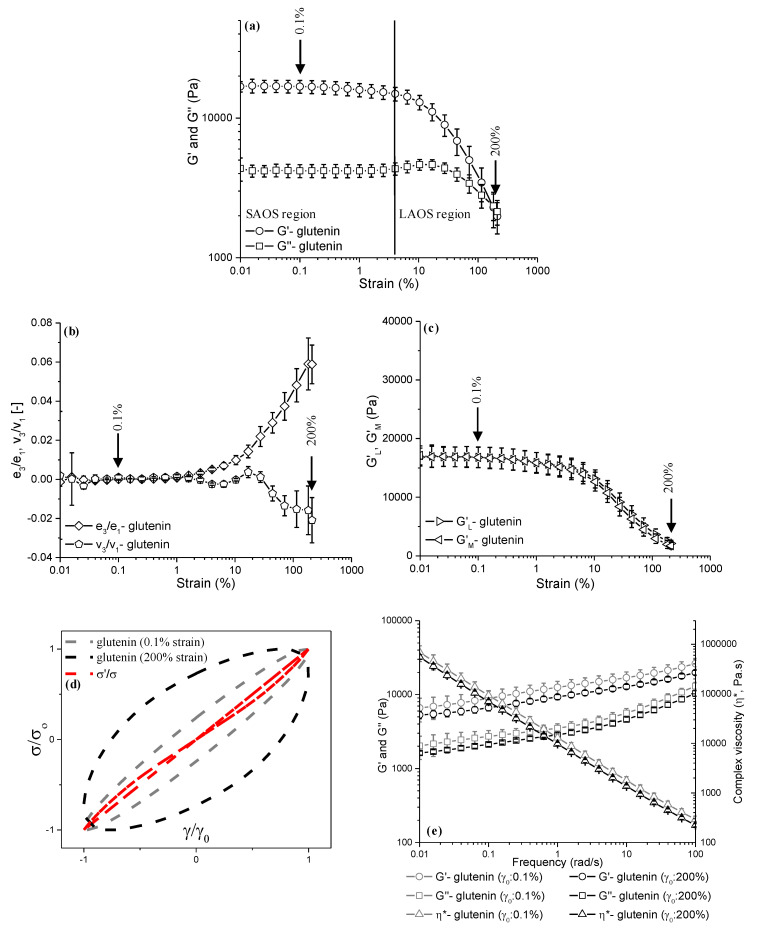
LAOS parameters and frequency sweep data for glutenin obtained at **60 min** of Farinograph mixing (γ: 0.1%, 200%): (**a**) LAOS sweeps, (**b**) the ratio of the third-order to the first order elastic (e_3_/e_1_) and viscous (v_3_/v_1_) Chebyshev coefficients, (**c**) large strain and minimum strain moduli (G_L_, G_M_), (**d**) elastic Lissajous-Bowditch curves at γ: 0.1% and 200%, (**e**) frequency sweeps.

The impacts of short-term (4 min) mixing (Figure 4) and prolonged (60 min) mixing (Figure 5), both followed by LAOS deformations of up to a γ_0_ of 0.1% and a γ_0_ of 200%, on the microstructure of glutenin were also evaluated through the strain sweep data (G′ and G″), LAOS parameters [elastic and viscous Chebyshev coefficients (*e_3_*/*e_1_*, *v_3_*/*v_1_*), large and minimum strain moduli (*G′_L_*, *G′_M_*)], elastic Lissajous–Bowditch curves, and frequency sweeps (G′, G″, η*, and tanδ).

The G′ values for the glutenin that underwent 4 min of Farinograph mixing (Figure 4a) were higher than those for the glutenin mixed for 60 min (Figure 5a) throughout the whole LAOS sweep. However, this decrease in the G′ of glutenin with respect to an increasing mixing time was not significant (*p* > 0.05). Thus, the LAOS sweeps indicated that the elastic response of glutenin both in the linear (γ_0_: 0.1%) and non-linear regions (γ_0_: 200%) was not affected by the increasing mixing time. The reason behind the insignificant decrease in the G′ values of glutenin with respect to an increasing mixing time could be due to the dissociation of the HMW and LMW glutenins with continued mixing, as reported by Bonilla et al. [12], leading to a decrease in the molecular weights of the glutenin molecules. Similar to gliadin, the G′ values of the glutenins also showed opposing trends to those of the Farinograph consistency (Figure 1b) as the mixing time increased. This conflict is attributed to the train regions of the HMW glutenins in the β-sheet conformation dominating over the loop regions in the β-turn conformation at the beginning of mixing [34], as seen in Figure 1d, which results in higher G′ values for the glutenin at 4 min of mixing (Figure 4a) in comparison to that obtained after 60 min of mixing (Figure 5a). However, due to the low hydration, the low number of hydration-induced extended structures, and the low number of crosslinks between the HMW and LMW glutenins in the early stages of mixing, the consistency of the glutenin is lower at 4 min of mixing (Figure 1b).

The onset of non-linearity occurred at a γ_cri_ of 4% for the glutenin regardless of the mixing time (Figure 4a and Figure 5a). Other studies have found similar results and reported the critical strain of glutenin as 2.5% [15] and 4% [20,44]. Unlike gliadin, glutenin displayed an overshoot at the onset of non-linearity only for G′′ beyond a strain value of 1.5% (Figure 4a and Figure 5a), while G′ decreased simultaneously. This observed behavior of the glutenin in the LAOS sweeps was characterized as type III non-linear behavior (weak strain overshoot) by Hyun et al. [40]. As the amplitude of the strain increased into the LAOS region, the G′ and G″ of the glutenin mixed for 4 min did not show any crossover points throughout the applied strain range. On the other hand, a crossover was observed for the glutenin mixed for 60 min at around a strain amplitude of 180%. These results indicate the reducing effect of prolonged mixing on the resilience of glutenin against increasing deformations, which is the opposite of what was observed for gliadin.

The *e_3_*/*e_1_* and *v_3_*/*v_1_* values started to depart from the margin beyond a γ_0_ of 2.5% for the glutenins mixed for 4 min (Figure 4b) and 60 min (Figure 5b), which concurred with the critical strain value suggested by the G′ and G″ data (Figure 4a and Figure 5a). As the applied LAOS deformation increased into the non-linear region, both the *e_3_*/*e_1_* and *v_3_*/*v_1_* values displayed a gradual increase, up to a γ_0_ of 200%. However, the magnitude of *e_3_*/*e_1_* for glutenin mixed for 60 min started to stabilize at the highest strain amplitudes applied (γ_0_: 180% and 200%), suggesting a halt in the increase in the intracycle strain-stiffening behavior of glutenin at high strain amplitudes when subjected to prolonged mixing. The magnitude of *e_3_*/*e_1_* at a γ_0_ of 200% was 0.060 ± 0.009 for the glutenin mixed for 4 min (Figure 4b), while it was 0.058 ± 0.009 for the glutenin obtained after 60 min of mixing (Figure 5b). These results suggest no significant difference in the intracycle strain-stiffening behavior of glutenin at a γ_0_ of 200% (*p* > 0.05) as the mixing time increases from 4 min to 60 min. The intracycle strain-stiffening behaviors of the glutenins mixed for 4 min and 60 min were also similar (*p* > 0.05) at lower strain amplitudes in the non-linear region, γ_0_ < 200%. On the other hand, the decrease found in the *v_3_*/*v_1_* values of the glutenin mixed for 4 min was steeper than that observed for the glutenin mixed for 60 min. At a γ_0_ of 200%, the *v_3_*/*v_1_* values were −0.035 ± 0.001 and −0.020 ± 0.011 for the glutenins mixed for 4 min and 60 min, respectively. However, this difference in the *v_3_*/*v_1_* values was not significant (*p* > 0.05), indicating that a prolonged mixing time does not affect the intracycle shear-thinning behavior of glutenin.

As observed for gliadin, the *G′_L_* and *G′_M_* values at 0.1% strain amplitude were almost identical (*p* > 0.05) for the glutenins obtained at both 4 min [Figure 4c; *G′_L_*: 27,569 ± 8511.6 Pa, *G′_M_*: 27,567 ± 8499.9 Pa] and 60 min [Figure 5c; *G′_L_*: 16,821 ± 1777.8 Pa, *G′_M_*: 16,803 ± 1741.4 Pa] of Farinograph mixing, as a γ_0_ of 0.1% (<γ_cri_) was within the limits of the linear viscoelastic region (Figure 4a and Figure 5a). As the LAOS strain increased to 200%, the *G′_L_* for the glutenin obtained at 4 min of Farinograph mixing was found to be slightly higher than the *G′_M_*, indicating intracycle strain-stiffening behavior (Figure 4c). On the other hand, the difference between the *G′_L_* and *G′_M_* values for the glutenin obtained at 60 min of Farinograph mixing with a γ_0_ of 200% (Figure 5c) was lower than that found for the glutenin obtained at 4 min of mixing (Figure 4c). The decrease in the difference between the *G′_L_* and *G′_M_* values for glutenin with respect to an increasing mixing time suggestes a lower degree of intracycle strain-stiffening behavior for glutenin when subjected to prolonged mixing, which is proven to not be significant (*p* > 0.05) by the elastic Chebyshev coefficients (Figure 4b and Figure 5b).

The normalized elastic Lissajous–Bowditch curves of the glutenins obtained at 4 min and 60 min of mixing are both narrow ellipses at a γ_0_ of 0.1% (Figure 4d and Figure 5d), which indicates the linear viscoelastic response of glutenin at a γ_0_ of 0.1%, concurring with the strain sweep data (Figure 4a and Figure 5a). As the applied LAOS strain increases to 200%, the elastic Lissajous–Bowditch curves show wider trajectories for the glutenin that underwent both short (Figure 4d) and prolonged mixing times (Figure 5d), suggesting a more viscous-like viscoelastic behavior under large deformations. Besides the wider elliptical shapes of the elastic Lissajous–Bowditch curves observable at a γ_0_ of 200%, the elliptical trajectory of the glutenin mixed for 4 min is distorted (Figure 4d) in comparison to that of the glutenin obtained at 60 min of mixing (Figure 5d). The distortion in the elastic Lissajous–Bowditch curves has been associated with intracycle strain-stiffening behavior [43]. Thus, the normalized elastic Lissajous–Bowditch curves at a γ_0_ of 200% indicate a decrease in the intracycle strain-stiffening behavior of glutenin as the mixing time increases, which is in line with the lower *e_3_*/*e_1_* (Figure 5b) and *G′_L_* and *G′_M_* differences found for the glutenin mixed for 60 min at a γ_0_ of 200% (Figure 5c).

Figure 4e shows the frequency sweeps for the glutenin obtained at 4 min of Farinograph mixing that was then subjected to 0.1% and 200% LAOS strains. The G′(ω) recorded for the glutenin at the lowest frequency (0.01 rad/s) was lower than that found at the highest frequency (100 rad/s), for both applied LAOS strains of 0.1% and 200%. In comparison to 4 min of mixing (Figure 4e), 60 min of Farinograph mixing resulted in lower G′(ω) values for the glutenin throughout the whole frequency range, with 0.1% and 200% LAOS deformations applied (Figure 5e). Although increasing the mixing time seemed to have reduced the G′(ω) of the glutenin versus the frequency, this decrease was not significant (*p* > 0.05), revealing no significant effect of increasing the mixing time from 4 min to 60 min on glutenin’s elastic response.

The highest η*(ω) values in the frequency sweeps were found for the glutenin that underwent 4 min of mixing followed by a 0.1% LAOS deformation (Figure 4e), while the glutenin subjected to 60 min of mixing and a 200% LAOS strain had the lowest η*(ω) values (Figure 5e). Thus, the stop-flow frequency sweep data revealed a decrease in the η*(ω) values of glutenin throughout the applied frequency range as the mixing time increased from 4 min to 60 min. However, this decrease was not significant (*p* > 0.05), concurring with the G′(ω) data.

The glutenin did not show a G′ = G″ crossover frequency and G′ predominated over G″ throughout the frequency sweeps, which were independent of the mixing time and the following LAOS strains applied (Figure 4e and Figure 5e). Such behavior is associated with a strong gel response [21], and indicates the elastic nature of glutenin.

### 3.4. Comparison of Microstructural Properties of Gliadin and Glutenin after Mixing and LAOS Deformations

**Figure 6:**
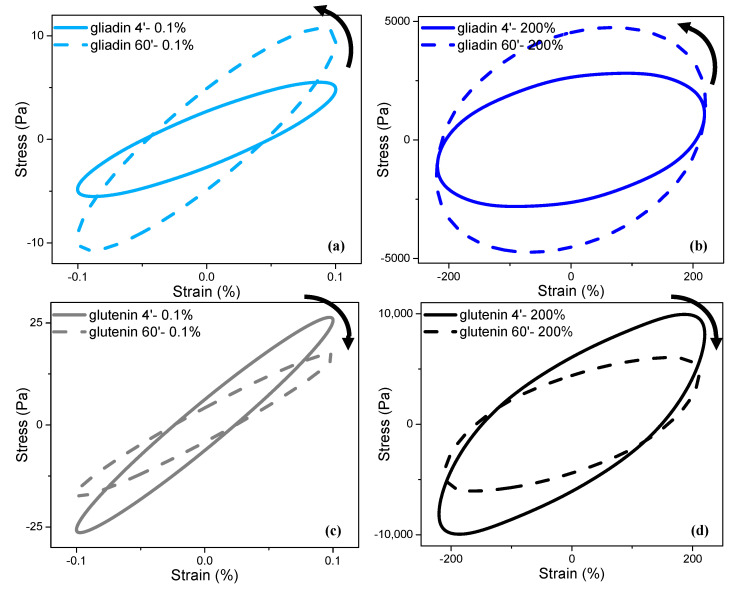
Raw elastic Lissajous–Bowditch curves for gliadin and glutenin subjected to short (4 min) and prolonged (60 min) mixing followed by two different LAOS strains: (**a**) gliadin at γ_0_ of 0.1%, (**b**) gliadin at γ_0_ of 200%, (**c**) glutenin at γ_0_ of 0.1%, and (**d**) glutenin at γ_0_ of 200%. Straight lines indicate 4 min of mixing; dotted lines indicate 60 min of mixing.

**Figure 7:**
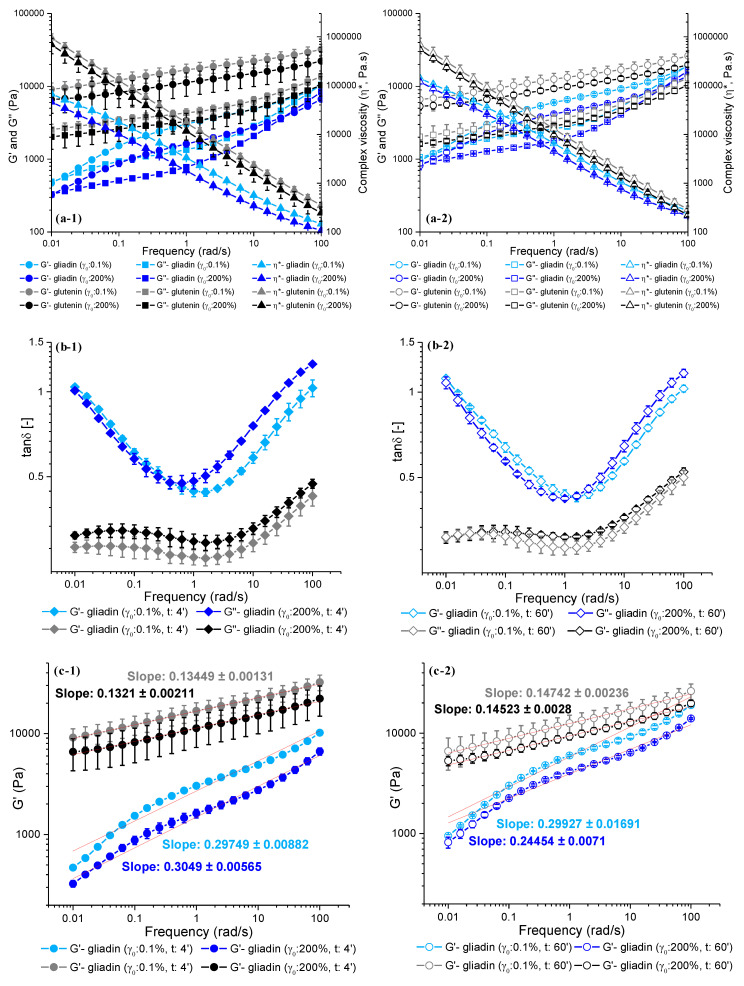
Impact of increasing strain amplitudes (0.1% and 200%) on microstructures of gliadin and glutenin obtained at short (4 min) and prolonged (60 min) mixing times: (**a-1**,**a-2**) G′, G″, and η* values versus frequency (0.01–100 rad/s), (**b-1**,**b-2**) tanδ versus frequency (0.01–100 rad/s), and (**c-1**,**c-2**) G′ slopes versus frequency. Numbers indicate mixing time: (**a-1**–**c-1**) 4 min and (**a-2**–**c-2**) 60 min.

The raw elastic Lissajous–Bowditch curves (Figure 6), obtained as part of the LAOS data, and the frequency sweep data (Figure 7) for the gliadin and glutenin are compared for an in-depth understanding of the impact of mixing and the following LAOS deformations on the microstructures of these main network-forming gluten proteins.

The raw elastic Lissajous–Bowditch curves highlighted the two opposite responses of gliadin and glutenin, both in the linear and non-linear regions, as the mixing time increased. The magnitudes of the maximum stress responses were higher (*p* < 0.05) for the gliadin that underwent 60 min of mixing in comparison to that mixed for 4 min, suggesting an increase in the elasticity of gliadin with prolonged mixing under both small (Figure 6a) and large deformations (Figure 6b). On the other hand, increasing the mixing time from 4 min to 60 min did not affect (*p* > 0.05) the magnitudes of the maximum stress responses of glutenin, which is indicative of the stability of glutenin under both small (Figure 6c) and large deformations (Figure 6d) after prolonged mixing.

The elastic Lissajous–Bowditch curves for gliadin show counter-clockwise rotation with an increasing mixing time (Figure 6a,b), while those for glutenin display clockwise rotation as the mixing time increases (Figure 6c,d), at all LAOS strains. The clockwise rotation of elastic Lissajous–Bowditch curves with increasing magnitudes of deformations has been associated with the softening of materials [43]. Thus, the elastic Lissajous–Bowditch curves indicate the intercycle stiffening of gliadin and the intercycle softening of glutenin as the mixing time increases from 4 min to 60 min. The G′ from the LAOS sweeps (Figure 2a, Figure 3a, Figure 4a and Figure 5a) and the maximum stress values of the raw elastic Lissajous–Bowditch curves (Figure 6) reveal that the intercycle stiffening of gliadin is significant (*p* < 0.05), while the intercycle softening of glutenin is not (*p* > 0.05).

The frequency sweeps conducted following the LAOS tests clearly showed an increase (*p* < 0.05) in the G′(ω) of gliadin and a decrease (*p* > 0.05) in the G′(ω) of glutenin as the mixing time increased from 4 min (Figure 7a-1) to 60 min (Figure 7a-2), which are in line with the elastic Lissajous–Bowditch curves (Figure 6). These results are the opposite of the Farinograph mixing results, which revealed a decrease in the consistency of gliadin (Figure 1a) and an increase in the consistency of glutenin (Figure 1b) as the mixing time increased from 4 min to 60 min. Thus, the Farinograph tests and the stop-flow LAOS–frequency sweeps revealed that the molecular organizations of gliadin and glutenin went through changes under mixing deformations and during the gap between the mixing and LAOS tests.

Increasing the mixing time significantly increased the G′, G″, and η*(ω) values of gliadin (*p* < 0.05) throughout the whole frequency range after the application of both LAOS strains. A higher η*(ω) was found for the gliadin mixed for 60 min in comparison to that mixed for 4 min (Figure 7a-1,a-2), while the Farinograms for gliadin indicated a reduction in consistency with prolonged mixing (Figure 1a), which could be due to the restoring ability of gliadin via intermolecular disulfide bonds under resting conditions. In earlier studies, the intrinsic viscosity of gliadin, measured before and after reduction and alkylation, showed that the viscosity values of gliadin under both conditions were similar, indicating that intermolecular disulfide bonds did not play any role in the conformation of gliadin. However, once reduced gliadin was reoxidized at a high enough concentration, an increase was observed in the viscosity, suggesting that intermolecular disulfide bonds, as well as intramolecular bonds, were formed [38,39]. At low and high frequencies, the impact of increasing the mixing time on the tanδ(ω) of gliadin was insignificant (*p* > 0.05), indicating that the G′ and G″ values were equally affected by low (0.01 rad/s) and high (100 rad/s) frequencies even if the gliadin was previously subjected to large deformations (Figure 7b-1,b-2). Increasing the mixing time significantly decreased (*p* < 0.05) the tanδ(ω) only for the gliadin subjected to a 200% LAOS strain at moderate frequencies (around 1 rad/s), indicating an increase in the elastic response due to the higher degree of increase in G′ than in G″. The crossover frequencies for gliadin (Figure 2e, Figure 3e and Figure 7a-1,a-2) also point to a more elastic response (G′> G″) at moderate frequencies (ω—0.012–45 rad/s for the gliadin subjected to 60 min of mixing and γ_0_—200%). These results reveal that prolonged mixing deformations and the following large LAOS deformations contributed to the elasticity of gliadin, which was mostly highlighted at moderate frequencies.

Mixing forces have been suggested to induce additional molecular interactions (i.e., water–protein, protein–protein) in wheat flour dough [7]. In the absence of water, HMW glutenin protein chains were thought to be hydrogen-bonded to each other, and these interchain hydrogen-bonded regions were suggested to be the train regions in a β-sheet conformation. As water was added to the glutenin, the number of water–protein hydrogen bonds, known as the loop regions in a β-turn conformation, started to increase [34]. At 4 min of Farinograph mixing, the glutenin was clearly in the early stages of hydration (Figure 1b), suggesting a higher proportion of trains to loops (Figure 1d). The possibly higher proportion of train regions in the β-sheet conformation of glutenin after 4 min of mixing (Figure 7a-1) was reflected in the frequency sweeps as higher G′, G″, and η* (*p* > 0.05) values when compared to those of the glutenin mixed for 60 min (Figure 7a-2), after the application of both LAOS strains. Increasing the mixing time from 4 min (Figure 7b-1) to 60 min (Figure 7b-2) and the LAOS deformation to 200% was the only condition that resulted in a significant increase in the tanδ(ω) of glutenin that became apparent at high frequencies (*p* < 0.05), indicating elastic decay. This can be attributed to the degradation of some glutenin molecules due to chain scission with prolonged mixing (Figure 1d) leading to a reduced molecular weight, as suggested by MacRitchie [36], and the dissociation of HMW and LMW glutenin subunits under continued mixing forces, as found by Bonilla et al. [12] in their co-localization studies, which became evident as the prolonged mixing was followed by large deformations (Figure 7b-1,b-2). In addition, the lowest tanδ(ω) throughout the whole frequency range was found for the glutenin subjected to 4 min of mixing followed by small deformations (γ_0_: 0.1%), indicating the highest elasticity for this sample (Figure 7b-1). Thus, the frequency sweep data point to the elastic nature of glutenin, which can be only reduced under prolonged mixing followed by large deformations and high frequency applications.

The glutenin mixed for 60 min (Figure 7c-2) had a higher G′ slope versus frequency, while the magnitude of G′ was lower when compared to that of the glutenin mixed for 4 min (Figure 7c-1) at both small and large deformations. The magnitude and the slope of G′ versus frequency are regarded as indicators of the degree of crosslinking [45]. Thus, the data in Figure 7c-1,c-2 suggest a reduction in the degree of crosslinking in glutenin when the mixing time increases from 4 min to 60 min. The impact of increasing the LAOS strain from 0.1% to 200% on glutenin’s viscoelastic response was lower, as it only caused a reduction in the magnitude of G′ versus frequency. On the other hand, the slope of G′ versus frequency for gliadin was only affected by the application of large deformations after prolonged mixing. A significant decrease in the slope of G′ versus frequency, along with an increase in the magnitude of G′ versus frequency, were observed when the gliadin underwent 60 min of mixing followed by large deformations (γ_0_: 200%) (Figure 7c-2), in comparison to the gliadin that underwent 4 min of mixing followed by a 200% LAOS strain (Figure 7c-1). This result revealed an increase in the degree of crosslinking in gliadin after being subjected to prolonged mixing and large deformations, which is consistent with the discussion of η*(ω), which suggested the formation of intermolecular disulfide bond formation in gliadin after prolonged mixing. The rheological tests conducted in the linear viscoelastic region on the gluten samples, to which different gliadin sub-groups were added, showed that the α-, β-, and γ-gliadins had higher degrees of crosslinking compared to the ω-gliadin subunits, indicated by their lower slopes and higher magnitudes of G′ versus frequency [14], which suggest that the α-, β-, and γ-gliadins could be responsible for the interactions contributing to the elasticity of gliadin under prolonged mixing. The fact that these gliadin subunits are also known to have a higher number of cysteine residues as compared to ω-gliadins [37] supports this result.

The slopes and magnitudes of the G′(ω) of gliadin and glutenin showed that the degree of crosslinking and elasticity was higher for glutenin under each deformation condition, and that the elastic response of gliadin approached that of glutenin’s when subjected to prolonged mixing followed by large LAOS deformations (γ_0_: 200%).

## 4. Conclusions

This study reveals the individual viscoelastic responses of the two main gluten fractions, gliadin and glutenin, which has never been done before, under short- (4 min) and long-term (60 min) mixing deformations followed by small (γ_0_: 0.1%) and large (γ_0_: 200%) LAOS strains through the application of stop-flow LAOS–frequency sweep tests.

The gliadin developed quickly after hydration and showed a peak at the early stages of Farinograph mixing (3.3 ± 0.5 min). However, the glutenin required more time to develop (31.4 ± 0.3 min), and continued to display development throughout the 60 min mixing process without showing a peak. These results reveal the contribution of gliadin to peak formation during wheat flour dough mixing, while glutenin supports the stability of the further stages of mixing.

The LAOS sweeps conducted after mixing indicated intracycle strain-stiffening and shear-thinning behavior for both the gliadin and glutenin under large deformations. Even though the literature suggests the origin of the strain-stiffening behavior of wheat flour dough is due to the entanglements of glutenin polymers, this study showed that gliadin also contributes to the strain-stiffening behavior of wheat flour doughs. Increasing the mixing time did not cause a significant difference in the intracycle strain-stiffening behaviors of gliadin and glutenin. On the other hand, prolonged mixing reduced the degree of the intracycle shear-thinning behavior of gliadin, while it did not influence the intracycle shear-thinning behavior of glutenin at large strain amplitudes.

Increasing the mixing time from 4 min to 60 min resulted in higher G′(ω), G″(ω), and η*(ω) values of gliadin at both LAOS strains. The slope of G′(ω) decreased for gliadin only after prolonged mixing followed by large LAOS deformations, indicating an increase in the degree of crosslinking. Thus, the stop-flow LAOS–frequency sweeps suggested that the mixing forces induced the development of the gliadin structure and improved the elasticity of gliadin, especially when followed by large deformations. The more elastic-dominated behavior imparted to gliadin by prolonged mixing could be due to protein aggregates with higher molecular weights possibly formed through intermolecular disulfide bonds during the gap between mixing and the stop-flow rheology tests. And, according to the slopes of G′(ω), this aggregation is further induced with the application of large deformations after prolonged mixing. On the other hand, increasing the mixing time of glutenin, followed by either small or large deformations, did not affect the G′(ω), G″(ω), or η*(ω). The stop-flow LAOS–frequency sweeps only captured an increase in the tanδ(ω) for glutenin when subjected to prolonged mixing followed by large LAOS deformations, which became apparent at high frequencies (*p* < 0.05). The elastic decay in glutenin, shown through the increase in tanδ(ω), could be attributed to the breakdown of the intermolecular disulfide bonds.

This new information obtained through Farinograph mixing and stop-flow LAOS–frequency sweep tests will help to shed light onto how these two major gluten proteins contribute to dough viscoelasticity during and after undergoing deformations. Further investigation is required to fully explore the chemical mechanisms behind the viscoelastic responses of the gluten proteins, which change in an opposite manner under a deformation. The fundamental approach used by this study could enable the precise optimization of processing parameters based on the viscoelastic properties of the gluten proteins in wheat flour for improved end-product quality. Thus, future studies may focus on correlating the individual linear and non-linear viscoelastic responses of gliadin and glutenin under different processing conditions with the quality characteristics of the end-products. The stop-flow LAOS–frequency sweep tests could also help to differentiate the processing quality of different wheat lines. In addition, the characterization of the linear and non-linear viscoelastic responses of other plant-based proteins, similar to that conducted for gliadin and glutenin in this study, could also lead to the optimization of formulas and processing parameters for the development of plant-based food alternatives with improved quality.

## Data Availability

The original contributions presented in the study are included in the article, further inquiries can be directed to the corresponding authors.
